# New Frontiers in Heart Rate Variability and Social Coherence Research: Techniques, Technologies, and Implications for Improving Group Dynamics and Outcomes

**DOI:** 10.3389/fpubh.2017.00267

**Published:** 2017-10-12

**Authors:** Rollin McCraty

**Affiliations:** ^1^Research, HeartMath Institute, Boulder Creek, CA, United States

**Keywords:** heart rate variability, social, coherence, self-regulation, synchronization, entrainment

## Abstract

Concepts embraced by the term coherence have been identified as central to fields such as quantum physics, physiology, and social science. There are different types of coherence, although the term always implies a harmonious relationship, correlations and connections between the various parts of a system. A specific measure derived from heart rate variability (HRV) provides a measure of physiological coherence. Another type of coherence, social coherence, relates to the harmonious alignment between couples or pairs, family units, small groups, or larger organizations in which a network of relationships exists among individuals who share common interests and objectives. A high degree of social coherence is reflected by stable and harmonious relationships, which allows for the efficient flow and utilization of energy and communication required for optimal collective cohesion and action. Social coherence requires that group members are attuned and are emotionally connected with each other, and that the group’s emotional energy is organized and regulated by the group as a whole. A number of studies are reviewed which have explored various types of synchronization in infants, pairs and groups, indicating that feelings of cooperation, trust, compassion and increased prosocial behaviors depends largely on the establishment of a spontaneous synchronization of various physiological rhythms between individuals. This article discusses a new application using HRV monitoring in social coherence research and the importance of physiological synchronization in group developmental processes and dynamics. Building on the extensive body of research showing that providing feedback of HRV coherence level at the individual level can improve self-regulation, we suggest the following hypotheses: (1) providing feedback of individual and collective HRV coherence and the degree of heart rhythm synchronization will increase group coherence, and heart rhythm synchronization among group members. (2) Training in techniques to increase group coherence and heart rhythm synchronization will correlate with increased prosocial behaviors, such as kindness and cooperation among individuals, improved communication, and decreases in social discord and adversarial interactions. (3) Biomagnetic fields produced by the heart may be a primary mechanism in mediating HRV synchronization among group members. Data supporting each of the hypothesis is discussed.

## Introduction

Research in evolutionary anthropology suggests that one of the primary drivers of evolution of the human species is our advanced capacities for social interaction and cooperation (1, 2). This research also suggests that humans are hard-wired to seek social connections and secure attachments, independent of maturational stage, and culture (3). Most people spend a sizeable amount of time communicating, interacting and performing tasks with others. Indeed, being a member of various groups across one’s lifespan is an inescapable, and often sought after, aspect of our lives. Some of our most precious moments are those of intimate connection. Terms such as social coherence, social support, social connection, social functioning, loneliness, and social isolation are used to describe various aspects of human social functioning. The importance of developing skills and behaviors and being able to successfully connect, cooperate and collaborate with others is of great importance.

Social incoherence not only affects the way we feel, relate, and communicate with others but also affects physiological processes that disrupt good health. In fact, when it comes to public health, social coherence and connection may be one of the most important public health priorities. The importance of social connections was highlighted by the sobering work of James Lynch who found that loneliness, itself, is a greater risk for heart disease than lack of exercise, smoking, excessive alcohol consumption, and obesity combined (4). Numerous studies have found that individuals experiencing social or cultural changes, or living in circumstances of social instability, disorganization, or isolation are at increased risk of acquiring numerous diseases (5–9). A meta-analysis of social relationships confirmed that when risk for mortality is controlled for based on well-established risk factors, there is a 50% increased likelihood of survival for people with healthy social relationships (10). Furthermore, numerous studies of various populations, regardless of geography or culture, have found that when individuals have close and meaningful relationships, they have reduced risk of mortality and susceptibility to chronic and infectious disease, have improved outcomes in pregnancy and childbirth (11–13) and live happier and healthier lives (10). The importance of social coherence for people’s health, as well as for team and organizational performance and societal harmony highlights the need for programs that strengthen people’s capacity for mental and emotional self-management and focus on increasing social coherence. Fortunately, to address what ails both individuals and groups in situations of social incoherence, there are practical steps and practices that can help increase and stabilize group coherence and resilience in organizations, teams, schools and communities.

In the context of facilitating social coherence, we (colleagues and collaborators) introduce following hypotheses: (1) Providing feedback of individual and collective heart rate variability (HRV) coherence and the degree of heart rhythm synchronization between group members will facilitate increases in the group’s coherence, and heart rhythm synchronization. (2) Training in techniques to increase group coherence and heart rhythm synchronization will correlate with increased prosocial behaviors, such as kindness and cooperation among individuals, improved communication, and decreases in social discord and adversarial interactions. (3) Biomagnetic fields produced by the heart may be a primary mechanism in mediating HRV synchronization among group members.

To support our hypotheses, we briefly review several types of coherence that are relevant to physiological processes and social coherence, and discuss the dynamic interplay between the two. We also explore emerging techniques and technologies to enhance social coherence. More specifically, we discuss a new application using HRV monitoring in social coherence research, and the importance of physiological synchronization in developmental processes and group interactional dynamics. Our primary hypotheses build on extensive research showing that providing HRV coherence feedback to facilitate skill acquisition of self-regulation techniques improves a wide range of health and performance outcomes. See Ref. (14, 15) for a summary.

## Coherence

As an overarching concept, coherence embraces several related phenomena that we will touch upon throughout the article: cross-coherence, entrainment, auto-coherence, and synchronization (14). Coherence always implies correlations, connectedness, consistency, efficient energy utilization, and typically refers to a global order, where the whole is greater than the sum of its individual parts.

In physics, cross-coherence is the term used to express the amount of synchronized activity among separate oscillating systems. When two or more systems have the same frequency range, they can become phase or frequency-locked as can occur in the human body when respiratory, blood pressure and heart rhythms, shift into an optimal state at the resonant frequency of the cardiovascular system (14). In this case, they are said to be entrained, however, different rhythms can be synchronized to varying degrees and not be entrained. Synchronization is an aspect of coherence that describes the coordination of distinct events that are acting in harmony whether concurrently or sequentially in an enduring patterned organization, between two or more events. In the context of social coherence, various mathematical measures of cross-coherence can be used to assess the degree of pairwise synchronization occurring between physiological rhythms, such as heart rhythms and brain waves of two or more individuals (16).

The term *auto-coherence*, sometimes called auto-correlation in mathematics, describes the order in a single waveform produced by an oscillating system, or the output of multiple complex interacting systems. For example, when the rhythm of the heart, exhibits sine wave-like output, the more stable the amplitude, frequency, and shape of the wave, the higher the degree of auto-coherence. When an oscillatory system has a high degree of auto-coherence and is coupled to other systems, it can pull the other systems into increased synchronization or entrainment, which reflects a more energy efficient and healthier system (17).

Thus, we use the term coherence in the broader sense to describe the degree of order, harmony, and stability in various rhythmic activities, which reflects the regulation of interconnected biological, social, and environmental networks. The assessment of physiological coherence (also referred to as heart coherence, resonance, cardiac coherence, or personal coherence) refers to auto-coherence (stability of a single waveform such as respiration or HRV patterns), and system resonance (18). In the context of this article, we will use the term synchronization to distinguish where we are referring to measures that are used to assess cross-coherence between pairs of individuals.

## HRV Coherence

The primary measure used to assess physiological or personal coherence is HRV. The most commonly used HRV analysis methods are frequency domain (power spectrums) and time statistical measures of variance in the inter-beat intervals (time domain), which are almost exclusively used to quantify the amount of HRV that occurred over a specific time period. As there are numerous reviews on the various measures of HRV and their underlying physiological mechanisms and interpretations, we will therefore not summarize them here (19–22).

Importantly, we have observed that specific emotional states are reflected in the patterns of the HRV rhythm as opposed to changes in the amount of HRV, and that emotions such as appreciation or compassion are associated with a more coherent rhythm, as opposed to emotions such as anxiety, frustration or impatience. “A coherent heart rhythm is defined as a relatively harmonic (sine wave-like) signal with a very narrow, high-amplitude peak in the low frequency region (typically around 0.1 Hz) of the power spectrum with no major peaks in the other bands (18). Coherence is assessed by identifying the maximum peak in the 0.04–0.26 Hz range of the HRV power spectrum, calculating the integral in a window 0.030 Hz wide, centered on the highest peak in that region, and then calculating the total power of the entire spectrum. The coherence ratio is formulated as: (Peak Power/[Total Power − Peak Power]^2^)” (22), p. 55. The association between heart rhythm patterns and emotional states has been demonstrated in studies conducted in both natural and laboratory settings (23–25). Thus, in order to assess physiological coherence in the context of one’s heart rhythm, the algorithm needs to detect changes in the pattern or structure of the HRV waveform, independent of how much HRV an individual may have.

## HRV and Self-Regulation

Porges (26) has proposed that the evolution of the autonomic nervous system (ANS), or more specifically the vagus nerve, was essential to the development of self-awareness and an expanded range of emotional experience. He also suggests that the vagal branch of the ANS in human beings plays a critical role in the social engagement system. Porges suggests that what he calls the social engagement system gives us the capacity for self-regulation and the ability to initiate prosocial behaviors when we encounter stressors, challenges or disagreements with others and that we are not limited to fight, flight, or freeze responses. He further suggests that the social engagement system acts as a “vagal brake,” and that assessment of vagal activity serves as an indicator of one’s ability to self-regulate emotions and behaviors. Therefore, lower levels of age-adjusted vagally mediated HRV reflect a low functional status of the social engagement system (26). This is supported by research indicating that higher overall levels of HRV is associated with psychological resiliency, behavioral flexibility and the ability to adapt to changing social demands (27). Furthermore, higher levels of resting state HRV are associated with better cognitive performance on tasks requiring executive functions (28).

## Social Coherence

Social or group coherence relates to pairs, families, groups or larger organizations in which there is a stable and harmonious alignment of relationships that allow for an efficient flow and utilization of energy and communication required for optimal collective action (14). Important aspects of group coherence include the degree of physiological synchronization, the capacity for coordinated action, and the number and quality of positive emotional connections, as well as harmonious cooperation, in the relationships of group members.

Individuals who comprise a working group, sports team, family or business organization may be aligned around a common purpose or goal and exhibit a natural tendency toward good communication, cooperation and efficiency (14). They can also share common, broader group identities such as cultural, religious, or political party affiliations and have to interact with individuals who belong to different groups, i.e., groups they are not aligned with. One of the significant challenges, however, in organizations and societies is that people tend to form biases and discriminate between “in-group” and “out-group” members, which can fuel tension, hostility, and unhealthy competition within the larger group (29). Such dynamics may affect families as well, to a lesser or larger degree. When social organization is incoherent and relations are discordant, optimal or even basic coordinated action may not be possible and psychosocial instability and dysfunction are likely consequences (30).

## Physiological Synchronization in Social Interactions

The smoothness or flow in social interactions depends largely on the spontaneous synchronization between individuals. When people are engaged in conversation, they unconsciously begin to synchronize their movements, vocal pitch, postures, speaking rates, and length of pauses between replies (31). As we are now discovering, important aspects of their physiology also can become synchronized. A number of studies have explored various types of physiological synchronization in infants, pairs, and groups.

In order for physiological activity of separate individuals to synchronize, a signal of some type (light, electromagnetic, sound, tactile, or chemical) must convey information between them. For example, the visual synchronization of physical movements increases feelings of affiliation (32), self-esteem (33), compassion and altruistic behavior (34), rapport (35), and increased prosocial behavior (36), while during arguments, synchrony decreases (37). In groups, synchronization has been shown to increase conformity (38), cooperation and trust (39), and the strengthening of social attachment between group members (39).

The importance of brain activity synchronization between individuals and in groups has recently gained interest in social neuroscience. However, the capacities for studying synchronization between brains during real-world social interactions are limited and tend to be methodologically clumsy, primarily due to the absence of suitable recording set-ups for simultaneous recordings of two or more people, and the lack of tools for multi-subject analysis.

The notion of brain-to-brain synchronization is that the perceptual system of one brain can be coupled to another brain (40). For example, using functional magnetic resonance imaging (fMRI), synchronization between a speaker and listener’s brain exhibit joint, temporally coupled response patterns during communication. Importantly, the more extensive the neural coupling between the speaker and listener pairs was, the more successful the communications was (41). The use of magnetoencephalography (MEG), which has a higher temporal resolution than fMRI, also demonstrated more rapid changes in the coupling of the listener’s and speaker’s cortical signals when interacting (42).

By using the electroencephalography (EEG), which is more practical than fMRI or MEG, in group settings, it has been possible to record multiple interacting participants’ brain synchrony and connectivity. For example, multiple participant EEG recordings have been obtained from musicians playing instruments together (43), during spontaneous nonverbal interactions (44) and to assess pairwise synchronization between students’ EEGs in a classroom setting (45). The Dikker study simultaneously recorded EEG activity from 12 students as they engaged in natural classroom activities and social interactions over 11 days and found that brain-to-brain synchrony between the students was consistently associated with increased class engagement and improved social dynamics.

## HRV as Measure of Interaction Synchrony

Another approach for evaluating interaction synchrony between individuals has done by looking at the synchronization of heart rhythms and HRV. HRV provides a means to evaluate ANS dynamics in real-time and does not practically or technically limit the number of participants that can be simultaneously monitored. As such, it provides an ideal approach for studying real-time dynamics during group interactions, as well as physiological synchronization. An important advantage is that HRV has the potential to reflect participants’ emotional states during various types of interactions. We can frequently identify state-specific patterns in the HRV waveforms that reflect real-time emotions, for example, frustration, anger, anxiety, and appreciation. These patterns are independent of the overall amount of HRV in an individual (18). Recent independent work has demonstrated 75% accuracy in discrete emotional state identification from the HRV waveform using a machine learning method for pattern recognition (25). The immediate reflection of emotional states in the HRV pattern is likely due to changes in the outputs of the subcortical structures involved in processing emotions as described by Pribram and Melges (46), Porges (26), and Thayer et al. (28) wherein the subcortical structures influence the oscillatory output of the cardiorespiratory centers in the brain stem. With HRV as a measure, we can simultaneously determine physiological synchronization, and the emotions occurring during the interactions.

## Mother–Infant Heart Rate and HRV Synchrony

Some of the earliest research utilizing HRV to assess interaction synchronization was on parent–infant synchrony and the coregulation of infant physiological process and emotional states through social interaction. Porges has suggested that the maturing of physiological oscillators provides the substrate for the regulation of the sleep–wake cycle, the heart rhythms, arousal, and the real-time registration of shifts in internal states such as hunger, blood pressure, and fatigue (47). Studies conducted by Feldman on infants’ biological clocks and cardiac rhythms from mid-pregnancy to term confirmed that the *in utero* development of an infant’s biological oscillator systems was critical for later synchronization with their mothers biological rhythms (48).

After birth, further work by Feldman, has shown that during face-to-face interactions, a mother unconsciously adapts her heart rhythms to those of her infant, and the infant adapts his or her heart rhythms to those of the mother in less than a second, resulting in a biological synchronization between the accelerations and decelerations of their heart rates (49). During periods of synchronization between the parent’s and child’s social interactions, the degree of synchronization between their heart rhythms increased, showing a real-time coordination between the physiological and social processes between separate individuals. The findings of this line of research led Feldman to suggest that biological rhythms provide the foundation for social rhythms (50).

Of further interest is that two forms of interactions were most effective in increasing synchrony between the HRV of mothers and infants: vocal synchronization and emotional state synchronization. The highest degree of HRV synchronization occurred when both emotional and vocal synchrony simultaneously took place, while gaze synchronization did not increase HRV synchronization. This synchronization did not require bodily contact or tactile stimulation as the mothers and infants were not in physical contact and rarely touched each other. Importantly, a more optimal organization or coherence of the biological rhythms between parents and the first months of their infant’s life was shown to predict the development of self-regulatory and social interaction capacities later in life (48, 51). Feldman et al. has suggested that the capacity to coordinate physiological rhythms may lie at the heart of human emotional connections and provides the foundation for collaboration and the formation of human societies (49).

## Energetic Fields and HRV Synchronization in Adults and Groups

Anyone who has experienced an exceptional concert or watched a championship sports team recognizes that something extraordinary can take place when a group surpasses its normal performance. At those times, it appears that the players are in-sync and communicating on an unseen energetic level. Many teams, such as professional and Olympic sports teams or Special Forces military units, appreciate and strive to increase team coherence although they may refer to it as “team spirit” or “bonding,” and often say then can “sense” a palpable “team energy.”

As mentioned earlier, in order for the physiological activity of separate individuals to synchronize, a signal of some type must convey information between them. In the previous section, research on the role of visual, auditory and tactile signals in mediating various types of synchronization between pairs was discussed. There are several lines of research suggesting that an energetic field connects individual group members directly, and which simultaneously distributes information between the group members. We have suggested that biologically generated magnetic fields may act as a carrier wave for information transfer between individuals and group members (52). The magnetic field produced by the beating heart, which is radiated externally to the body, provides a plausible mechanism for conveying information to locations external to the body and for how some people can “feel” or “sense” another person’s presence (53) or emotional state, independent of body language, or other factors (16).

An interesting study that is also suggestive of an information field connecting group members was conducted during a fire-walking ceremony that looked at synchronized heart rhythms between firewalkers and spectators (54). During a 30-min ceremony, they found a high degree of synchronized activity between the firewalkers and spectator’s HRV who had an emotional connection to them. The unrelated spectators who did not have an emotional connection to the firewalkers did not have any HRV synchronization with the firewalkers. The researchers concluded that the mediating mechanism must be information that was somehow distributed among the group members. A number of other investigators have found results that are consistent with this hypothesis. For example, a study examining HRV synchronization and coherence levels in 10 groups, each with four individuals, found that being in an HRV coherent state helped others to shift into a more coherent state (55). They also found that HRV synchronization between participant pairs was increased during periods of increased individual HRV coherence and was correlated with the degree of emotional bonding between participant pairs. The authors concluded that “evidence of heart-to-heart synchronization across subjects was found which lends credence to the possibility of heart-to-heart bio-communications.”

In a study investigating physiological synchronization during nonverbal compassionate communication, Kemper and Shaltout found significant changes in the receiver’s ANS (56) that was correlated with the sender’s. A study by Russek and Schwartz also found that cardiac-related information exchange occurs between individuals and that the amount of physiological synchronization between pairs was greater in people raised in a loving environment. They found that the EEG of one person synchronizes to heartbeats (ECG) of another person sitting across from them. Participants who had rated themselves 40 years before the study as having grown up in a loving environment, had significantly more heart–brain synchronization between the pairs than individuals who reported not being raised in a loving environment (57).

**Figure 1:**
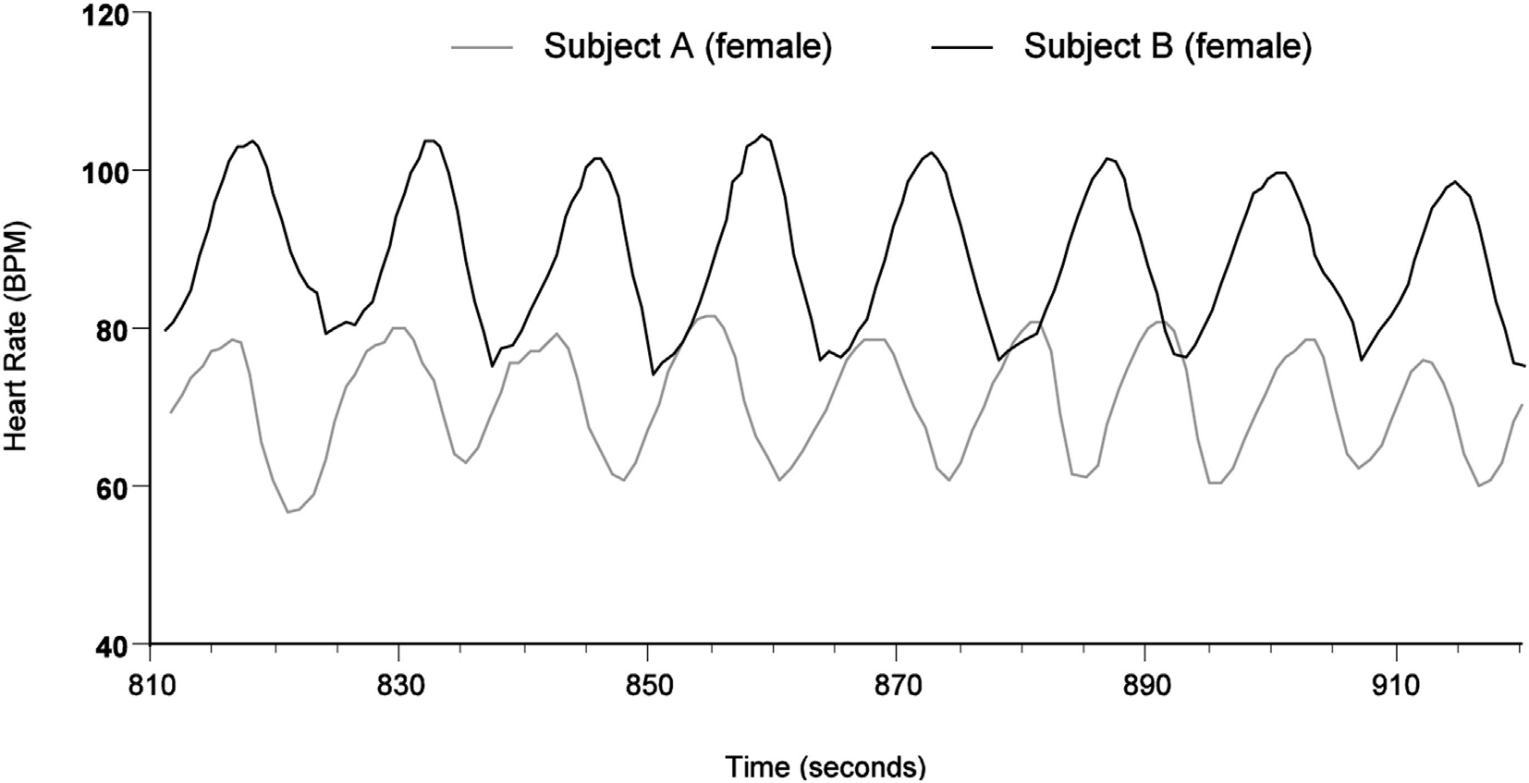
Heart rhythm entrainment between two women. The data were recorded during a period while both participants were consciously feeling appreciation for each other.

In our laboratory (16), we have observed that entrainment (phase or frequency locking) of the HRV patterns between individuals, is rare during normal waking states and that people who have a close working relationship or live together in a bonded relationship are the best candidates for exhibiting this form of HRV synchronization. Figure 1 shows an example of heart rhythm entrainment between two women who have a close working relationship and who were seated 4-feet apart with their backs to each other and with their eyes closed while they were feeling appreciation for each other.

**Figure 2:**
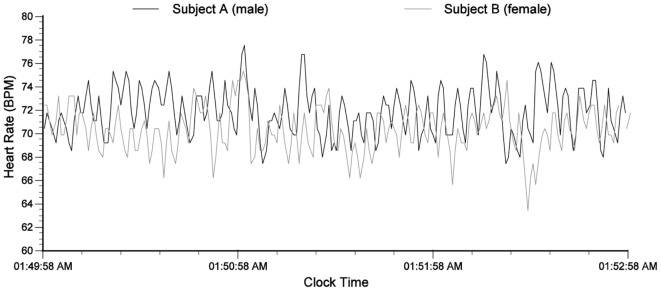
Heart rhythm synchronization between a husband and wife during sleep.

Heart rate variability synchronization can also occur during sleep between couples who are in a stable and loving relationship, although entrainment of the two rhythms may not be present as shown in Figure 1. Figure 2 shows an example of a 3-min segment of data from one such couple. Note how the HRV rhythms concurrently change and how heart rates converge.

As with the firewalkers, these examples suggest that a positive emotional connection between people is an important factor in heart rhythm synchronization. Further support for an energetic connection across group members and the importance of positive emotional connections also emerged from research conducted by Raymond Bradley and Karl Pribram. They developed a theory of social communication to explain the arrangements of social organization common to many groups, independent of size, culture, and degree of formal organization (58). By studying numerous groups, they found that most groups have a global organization, structured as a coherent network of emotional connections. By mapping the self-reported relationships between all of the possible combinations of pairs in a given group, they found a strong relationship between the number and structure of reciprocated positive emotional bonds and the structure of the control relationships among group members that predicted group stability and performance 2 years later (59). The theory that best fit the data was one constructed on a field concept where information about the structure of the entire group was simultaneously distributed to all of the group members, a collective consciousness, which they called a “social hologram” (58).

## The Heart’s Magnetic Field

**Figure 3:**
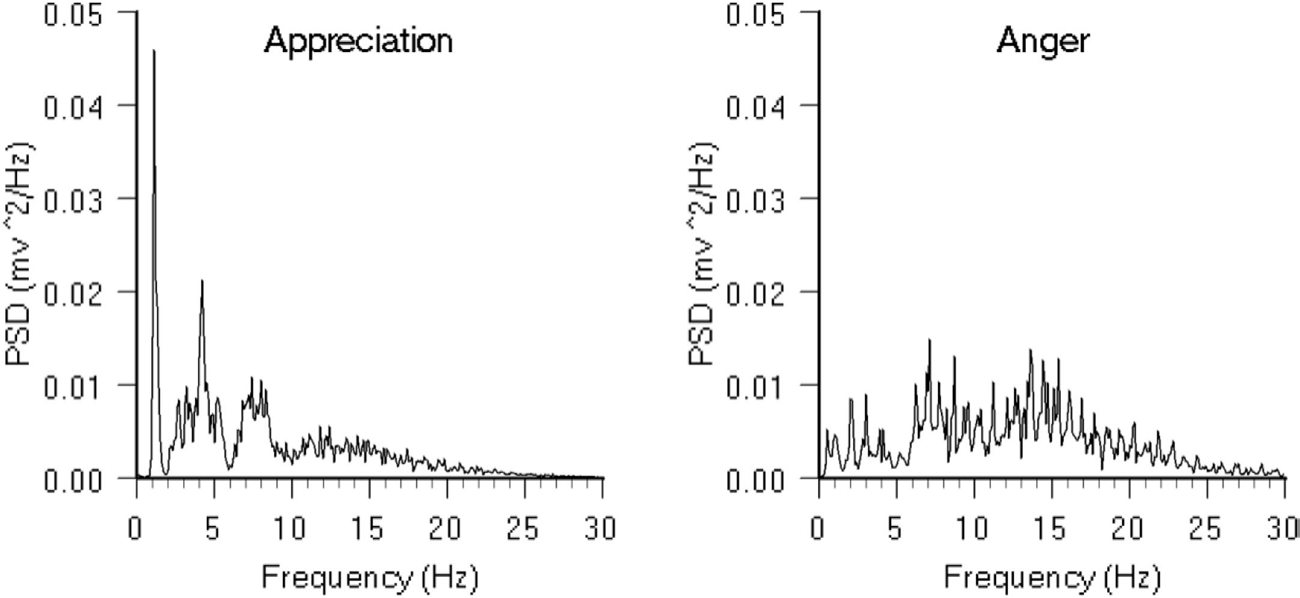
ECG spectra during different emotional states. The above graphs show the average power spectral density (PSD) of 12 individual 10-s epochs of ECG data. The graph on the left is an example of a spectrum obtained during a period of high heart rhythm coherence generated during a sustained heartfelt experience of appreciation. The graph on the right is the spectrum of a disordered heart rhythm recorded during feelings of anger. It can be clearly seen that the spectral patterns in the magnetic fields radiated by the heart have different structures and informational content.

The various explorations discussed above suggest that a biomagnetic communication system may exist which serves to connect and distribute information among members of stable groups. In the human body, the heart generates the largest rhythmic magnetic field, one that is about 100 times the amplitude produced by the brain. This field, which is measured in the nanotesla range, can be measured several feet outside the body with SQUID-based magnetometers (60). We have found that information reflecting one’s emotional state not only is encoded in the patterns of the HRV waveform but also is contained in the heart’s electromagnetic field radiated into the environment (16, 18). When an individual is in a heart coherent state, the heart’s magnetic field also has a more coherent structure (Figure 3). There is a direct mathematical relationship between the HRV patterns and the spectral information encoded in the magnetic field (18).

The heart radiates a series of pulsing magnetic waves, in which the time interval between each pulse of magnetic energy varies in a complex manner. These pulsing waves of magnetic energy produce interference patterns when they interact with magnetically polarizable tissues and substances (18). For example, we have shown that the heart’s magnetic field can be detected by the nervous systems of nearby people or animals (16).

Our hypothesis is that biomagnetic fields produced by the heart may be a primary mechanism in mediating HRV synchronization among group members. This perspective is supported by the work of quantum physicists Larissa Brizhik and Emilio Del Giudice. They have suggested that magnetic fields are the most likely physical agent that can continuously provide an exchange of information between living systems within the larger ecosystem. They specifically suggest that magnetic potentials provide the mechanism for establishing self-consistency, coherence, complexity, and non-local information exchange found in any living system and ecosystems (61, 62).

The messengers should be the electromagnetic fields produced by all the coherent parts of the organism. We recall that a coherent system is one where the phase (namely the rhythm of oscillation of the coupled matter and electromagnetic field) is sharply defined. Here we generalize this approach to a larger scale and show that according to quantum field theory, the electromagnetic field is the messenger that, *via* its electromagnetic potential, governs the dynamics of not only individuals, but of the whole ecosystem to which the individuals belong. This generalization is based on the fact that the field causes the emergence of the coherent structures, which, in view of their coherence, openness and nonlinearity, are able to self-organize and form a chain of hierarchical levels of ecosystems (61) (p. 1856).

## New Frontiers

### Development of a Group HRV Assessment Platform

There is an extensive body of research showing that providing HRV coherence feedback to facilitate skill acquisition of self-regulation techniques improves a wide range of health and performance outcomes (15, 63–86).

As stated in the Section “Introduction,” one of our hypotheses is that providing feedback of individual and collective HRV coherence and the degree of pairwise heart rhythm synchronization will facilitate increases in the group’s coherence and heart rhythm synchronization among group members. We are further suggesting that providing HRV coherence feedback to groups about the group’s coherence as a whole will help encourage individuals in the group to better self-regulate and engage in more prosocial behaviors. We also propose that it will encourage group members of families, schools, organizations, and communities to learn how to get more in-sync with each other before and during meetings and important communications, while making important decisions or when individuals need to resolve a conflict, etc.

We are in the process of developing new techniques, exercises, and processes for building and sustaining group coherence, which build on the self-regulation techniques used for achieving personal coherence, discussed in the next section.

As discussed above, the experimental paradigms to evaluate the physiological aspects of social interaction have been limited. Typical experiments tend to isolate people from their natural surroundings by placing them in a sealed room where interactions take place through computer games and video or auditor links. Many, if not most social interactions such as meetings, conversations, and other group engagements take place face-to-face and depend on real-time perception of social signals and various forms of physiological synchronization. Given the practicality of recording HRV during group interactions and its usefulness in understanding real-time ANS dynamics, stress and emotional states, and pairwise synchronization among group participants, we have developed a new research system for simultaneously measuring the HRV of group members. The experimental control software provides real-time feedback of group’s collective coherence level and degree of HRV synchronization among all the possible pairwise combinations. Each participant is provided with feedback of their own HRV coherence level while simultaneously being presented with a representation of the group’s overall coherence score. In other words, there are two types of coherence that are assessed and displayed to the group members. The group’s average coherence score (calculated with the algorithm described in the physiological coherence section above) and degree of pairwise HRV synchronization between members grouped into various clusters are presented. We are currently testing several analytic approaches for assessing pairwise synchronization, as well as the most effective approaches for providing feedback to the group members. For example, we have developed and validated a method using near-optimal chaotic attractor embedding techniques to identify specific patterns and clusters of HRV synchronization between people which is described elsewhere (87).

We have also developed custom pulse wave data acquisition modules that allow for analysis of the pulse waves which yields information that complements the information provided by analysis of the HRV waveform, such as changes in arterial wall stiffness, which is affected by sympathetic system activations (88, 89). While currently configured to simultaneously monitor 20 participants, there are no limits on capacity as the system is designed to be easily be scaled up to any number of channels. The system uses an ethernet connection to link the host computers, and considerable effort was invested in time stamping each data channel so that accurate timing of the data collected from each participant is maintained. This allows the group members to be located anywhere in the world as long the host computer has an internet connection.

One of the planned future additions to the system is voice analysis capability which will allow for a more detailed analysis of events that are associated with changes in the HRV during verbal interactions in environments such as business or classroom settings. It will be possible to determine when people are talking over others in meetings and extract information relating to the emotional state of each speaker through spectral analysis of each participants’ speech. This will augment the considerable amount of emotional content already detectable in the HRV alone (25). It is also our intention to implement a machine-learning based assessment of emotional state recognition and detection from the HRV and voice signals to aid in detecting pattern in the group dynamics.

The group HRV coherence research system is also designed to interface with the easily available laptop-based emWave Pro (HeartMath, Inc., Boulder Creek, CA, USA) HRV coherence training device which will provide a low cost option for researchers interested in doing group-related HRV experiments. We also plan to develop the capacity for the system to connect to Bluetooth devices such as the Inner Balance Trainer for mobile devices, as well as others, and to develop at least one game in which success is achieved through the combined ability of a group’s members to sustain coherent HRV states.

**Figure 4:**
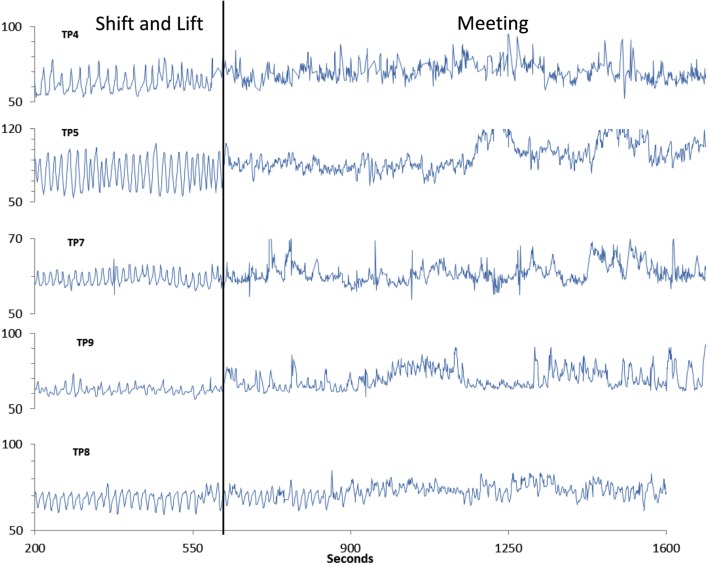
An example of five participants who were simultaneously recorded while using the Shift and Lift technique for approximately 5-min before the start of a business meeting (left side). The second segment is a 15-min period during the meeting. It can be clearly seen that while the participants were using the Shift and Lift technique that four of the five participants were in a more coherent state than during the meeting.

As of summer 2017, we are collecting data during a number of different types of group interactions, such as business meetings, interactive game play, and various heart-based meditations. This research meets all the applicable standards for the ethics of experimentation in accordance with the Declaration of Helsinki, and participants provided written consent prior to participation in data collection. Figure 4 shows an example of data collected from a group of five individuals seated at a conference table while using a newly developed technique as part of the training program for increasing social coherence called Shift and Lift which is intended to be used before communicating with others. This example is included only to provide a visual reference for the difference between a coherent heart rhythm as occurred in the first segment labeled Shift and Lift, and the following segment, where the individuals were engaged in a business meeting, where there was not much HRV coherence. It can clearly be seen that during the business meeting segment, that each had much less HRV coherence and there was not any obvious pairwise synchronization between the individuals.

**Figure 5:**
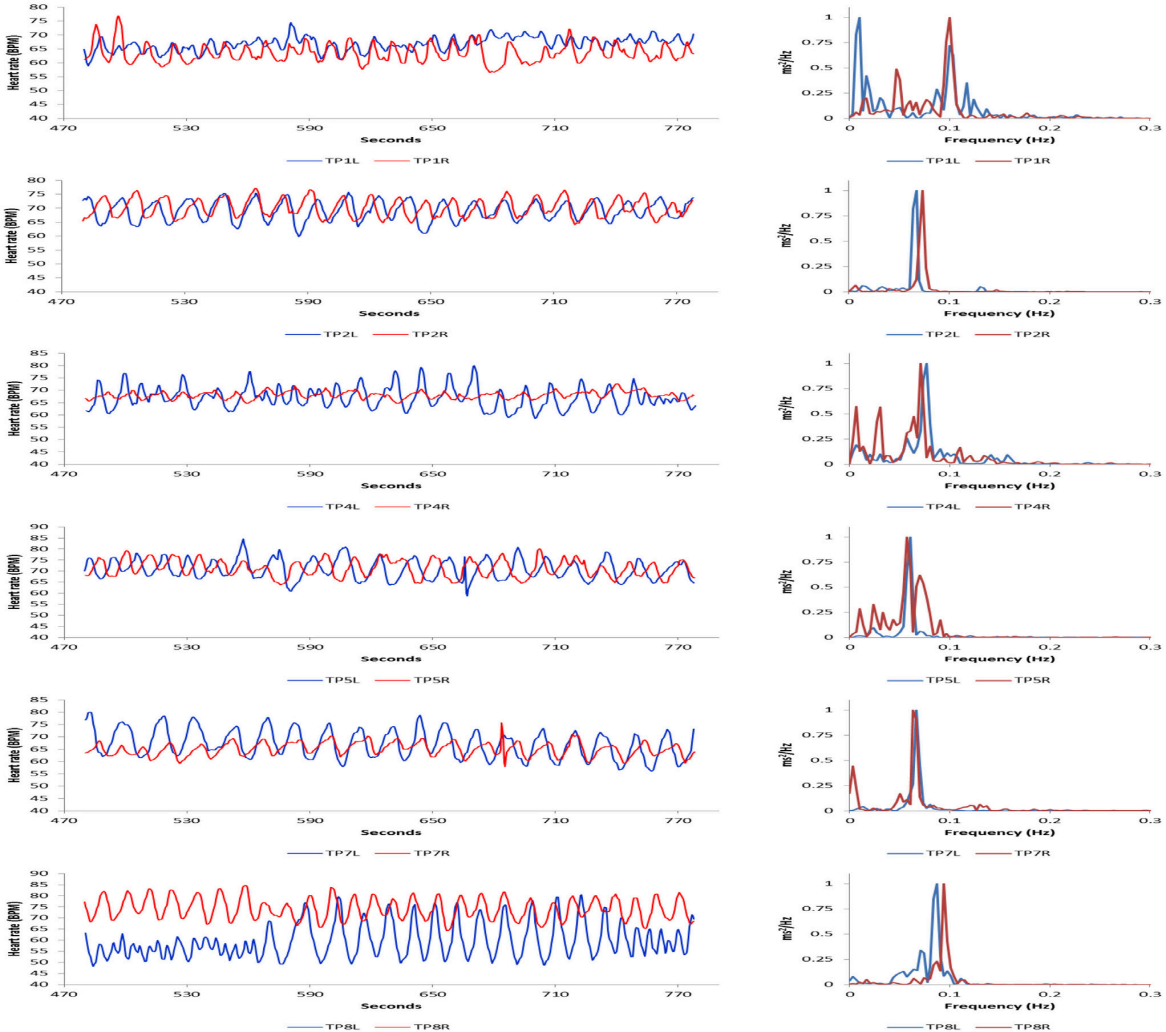
An example of 12 participants who were simultaneously recorded while using the Heart Lock-In technique for 5 min. The pairs were instructed to actively feel and radiate feelings of appreciation to the other pair partner. The left hand side shows an overlay of the HRV waveforms for each pair of participants, while the right hand side shows an overlay of the HRV power spectrums of each pair.

Figure 5 shows an example of 12 participants who were simultaneously recorded.

While they were seated around a long conference table and instructed to use the Heart Lock-In technique for 5 min, which is a heart focused meditation like technique. All of the participants were experienced in using the technique and known from previous experiments to be able to shift into and sustain a coherent HRV rhythm. During this experiment, the pairs were preassigned and instructed to keep their eyes closed during the Heart Lock-In while focusing on actively radiating feelings of appreciation to the other pair partner. The left side of the figure shows an overlay of the HRV waveforms of each pair of participants. The right side of the figure shows an overlay of the HRV power spectrums of each pair. Preliminary results suggest that there is increased HRV synchronization between participant pairs when they focus on radiating positive feelings specifically toward each other, as opposed to a more general focus, such as radiating positive feelings or intentions to the people in a remote location, or all the members of the group. The figure is included only as an example of how the platform allows for easier multi-subject data collection than was previously possible, although this example did not utilize some of the other unique features of the platform, such as real-time group coherence feedback, or feedback on pairwise HRV synchronization.

## Training Programs to Increase Social Coherence

In most social contexts, individuals at times form preconceptions or judgments toward one another and hold onto feelings that are often unspoken, but that leads to poor communication and other damaging social dynamics that disrupt optimal team or group performance. Our second hypothesis states that training in techniques to increase group coherence and heart rhythm synchronization will correlate with increased prosocial behaviors, such as kindness and cooperation among individuals, improved communication, and decreases in social discord and adversarial interactions.

Numerous large-scale implementations of training programs for increasing personal self-regulation skills and resilience in hospitals, military and law enforcement, educational, and business environments which included HRV coherence feedback support the hypothesis. It has been shown that providing emotional self-regulation skills combined with heart rhythm coherence training results in significant improvements in communication, employee satisfaction, productivity, problem solving, reduced turnover, and a significant return on investment, both financially and socially (15, 67, 73, 86, 90–92). In our experience, the self-regulation and resilience building training programs are most successful when leadership openly models and support the programs (93). These programs include practical skills that increase self-regulatory capacity and physiological coherence as a key element. Another important lesson, perhaps the most important, is the essential need to implement a robust sustainability strategy. No matter how effective the techniques, exercises, processes or technologies may be for self-regulation and group coherence, their effectiveness cannot be realized if they are not utilized on a consistent basis. As with any new skill, it takes repetition and practice before it becomes routine, especially during challenging situations, when they are needed most. In our experience, the most effective approach to facilitate the ongoing use and grounding of the skills is by providing participants with ongoing support from a team mentor or coach. The main objective of the mentoring is to provide the team members and leadership with continued support, knowledge and tactics to effectively expand and sustain self-regulation and social coherence skills and practices.

We are in the process of developing new techniques, exercises and processes for building and sustaining group coherence, which build on the self-regulation techniques used for achieving personal coherence such as the Heart Lock-In and Freeze Frame, techniques (94–96). It requires effort and energy to shift an incoherent group into a more coherent mode, and an important key is the establishment of more positive emotional bonds, and dissipating negative emotional tensions, interpersonal conflicts and other stressors within the individuals that comprise the group. The newly developed techniques and processes focus on issues such as clearing historical judgments and misunderstandings, appreciating differences, aligning with core values, coherent communication, improving the emotional climate, understanding energetic field environments, avoiding the pitfalls of “groupthink” and utilizing the power of positive “emotional contagion.” The majority of the techniques we employ in the social coherence building intervention are designed to be used in the moment one is “triggered” or is experiencing emotional undercurrents or stress. Several of the techniques are designed to be used by groups to “get coherent” and be better prepared for upcoming events that may be challenging such as meetings or interactions with customers or vendors who have a history of being difficult to work with.

We will be conducting a series of field research studies in various groups to refine the social coherence training program content and to evaluate the effectiveness of the HRV based group coherence platform for increasing social coherence. The essence of the hypothesis is that the combination of HRV coherence assessment and feedback at the group level with an educational program focused on increasing a group’s coherence will result in a number of benefits. We predict that there will be an increase in positive energy and bonding, care, kindness and cooperation among individuals, creativity and decision-making, appreciation of group members’ differences, improved communication, and a decrease in social discord and adversarial interactions resulting in shorter meeting times, fewer mistakes, increased academic performance in schools and increased sense of well-being and collective purpose. In order to assess these types of outcomes, we are also in the process of developing a psychometric assessment specifically for the key constructs of group harmony and coherence.

## Conclusion

Social coherence was defined as a stable, harmonious alignment of relationships, which allows for the efficient flow and utilization of energy and communication required for optimal collective cohesion and action. Various studies examining synchronization between mothers and infants, pairs and groups, indicate that feelings of cooperation, trust, compassion and prosocial behaviors are facilitated by physiological synchronization between individuals.

Building on the extensive body of research showing that providing feedback of one’s HRV coherence level at the individual level can improve self-regulation, we suggest providing feedback of individual and collective HRV coherence and the degree of heart rhythm synchronization will increase group coherence and heart rhythm synchronization among group members.

The development of a new HRV recording system allows for the real-time assessments during group interactions and provides real-time feedback on group dynamics, collective group coherence levels and HRV synchronization among all the possible pairwise combinations. The occurrence of HRV synchronization between clusters of group members has been demonstrated and has been shown to be associated with positive emotional bonds between group members.

We also discussed support for the hypothesis that training group members self-regulation techniques designed to increase group coherence and pairwise HRV synchronization will correlate with increased prosocial behaviors, such as kindness and cooperation among individuals, improved communication, and decreases in social discord and adversarial interactions. Our hope is that this new technology and training program will decrease social discord and increase positive emotional connections, kindness, cooperation, overall social well-being, and sense of collective purpose.

## Ethics Statement

The research met all applicable standards for the ethics of experimentation in accordance with the Declaration of Helsinki. Participants provided written informed consent prior to participation in the study.

## Author Contributions

The author confirms being the sole contributor of this work and approved it for publication.

## Conflict of Interest Statement

RM is employed by the HeartMath Institute which is a non-profit research center supported by grants, donations and some fee-for-service activities such as providing resilience trainings, sales of books and heart rhythm coherence technologies, all of which is focused on services to education, service members, veterans, and non-profit social services agencies. The HeartMath Institute does not manufacture any devices, and if they are included in research projects or resold, are purchased from the manufacturer in the same way as any other organization.
